# Iron metabolism and chronic inflammation in IgA nephropathy

**DOI:** 10.1080/0886022X.2023.2195012

**Published:** 2023-04-04

**Authors:** Zhang-yu Tian, Zhi Li, Ling Chu, Yan Liu, Jin-rong He, Yu Xin, Ai-mei Li, Hao Zhang

**Affiliations:** a Department of Nephrology, The Third Xiangya Hospital, Central South University, Changsha, China; b Department of Pathology, The Third Xiangya Hospital, Central South University, Changsha, China

**Keywords:** IgA nephropathy, iron metabolism, chronic inflammation, ferroptosis, treatment

## Abstract

IgA nephropathy (IgAN), an immune-mediated chronic inflammatory kidney disease, is the most common primary glomerular disease in Asia, especially in China and Japan. The pathogenesis of IgAN is complex, and the main cause of IgAN is explained by the ‘multiple hit’ theory, which states that the deposition of immune complexes in renal mesangial cells induces chronic inflammation that leads to kidney damage. Chronic inflammation is associated with iron metabolism, which also plays an essential role in the pathogenesis, progression, diagnosis and prognosis of IgAN. Overall, this review aimed to explore the application of iron metabolism in IgAN by systematically elaborating the relationship between iron metabolism and chronic inflammation in IgAN to speculate on the possible diagnostic and therapeutic significance of iron metabolism indicators in IgAN.

## 1. Introduction

Iron is an indispensable element. Iron is regulated at the systemic level and the cellular level, mainly through the absorption, transport, distribution, storage, utilization and excretion of iron metabolic processes to maintain iron homeostasis in the human body [1]. Iron metabolism disorders are closely associated with a diversity of diseases, such as cancer, neurodegenerative diseases, ischemia–reperfusion injury, hematologic disorders, inflammatory bowel diseases, liver diseases, pulmonary fibrosis and chronic kidney disease (CKD) [2–9]. Additionally, the kidney influences the regulation of iron homeostasis [10–12], while imbalances in iron homeostasis can exacerbate kidney injury [11,13–15].

IgAN is the most common primary glomerulonephritis in the world, and approximately 30% of IgAN patients develop end-stage renal disease (ESRD) within 20 years [16,17]. IgAN accounts for approximately 40% of all natural kidney biopsies in Japan, 25% in Europe, and 12% in the United States but less than 5% in Central Africa [18]. IgAN is a chronic inflammatory disease, and its pathogenesis and progression are associated with many indicators of iron metabolism [19–21]. In addition, IgAN is characterized by hematuria and proteinuria as the main clinical manifestations [22]. Iron from hemoglobin metabolism in hematuria is toxic to proximal tubular cells [23], mainly because of the ability of renal tubular epithelial cells to phagocytose and degrade red blood cells [24–26], leading to an increase in Fe^2+^, which reacts with H2O2 to produce reactive oxygen species (ROS) to damage kidney cells [27]. Thus, IgAN, iron metabolism, and chronic inflammation are related and have a complex relationship. When elaborating this relationship, we need to focus on the importance of iron metabolism in IgAN and increase research on the mechanism of hematuria severity with IgAN.

## 2. Physiological iron metabolism

### 2.1. Body iron homeostasis

Iron metabolism is regulated at the systemic and cellular levels. Systemic iron homeostasis is mostly adjusted by the hepcidin-/ferroportin axis [28,29]. Because iron losses are relatively small, iron absorption and its regulation by hepcidin and ferroportin (FPN) determine systemic iron levels [30]. FPN exports iron from duodenal enterocytes that absorb dietary iron, from iron-recycling macrophages in the spleen and the liver, and from iron-storing hepatocytes [31]. Hemoglobin in mammals contains more than half of the total human iron content [32]. Hemo Oxygenase-1 (HO-1) degrades hemoglobin to bilirubin, Fe^2+^ and carbon monoxide, and the released iron can be exported from macrophages *via* FPN or stored intracellularly *via* ferritin [33,34]. Hepcidin is a negative regulator of iron metabolism that blocks iron export through FPN [30]. When iron deficiency or hemorrhage occurs, hepcidin decreases to allow iron delivery to plasma through FPN, promoting compensatory erythropoiesis. During infection or inflammation, hepcidin blocks iron delivery to plasma *via* FPN, limiting the supply of iron to invading microorganisms [29]. Cellular iron levels are largely modulated by the iron response element-iron regulatory protein (IRE-IRP) system, which adjusts the expression of transferrin (Tf), transferrin receptor 1 (TfR1), divalent metal transport protein 1 (DMT1), FPN, and ferritin [35]. When the cellular iron concentration is relatively low, IRP binding to IRE of TfR1 and DMT1 promotes the expression of both and augments iron uptake; conversely, IRE is occupied by Fe/S proteins, which prevents IRP from binding to IRE of TfR1 or DMT1, resulting in lower levels of TfR1 and DMT1 translation and less iron uptake when cellular iron levels are comparatively high [36]. Under iron loading conditions, this eventually leads to the over-saturation of the iron carrier protein transferrin and the generation of non-transferrin bound iron (NTBI)[37]. A growing body of literature indicates that NTBI uptake is mediated by non-transferrin-bound iron transporters such as ZIP14, L-type and T-type calcium channels, DMT1, ZIP8, and TRPC6 [38–42].

### 2.2. Renal iron homeostasis

Three regulatory mechanisms are relevant to iron metabolism in the kidney, including the IRE-IRP system, HIF regulatory system and renal reabsorption [11,12,43–48]. Many transport proteins and regulatory pathways involved in cellular iron handling have been identified in the kidney [10,45,47,48]. IRP1 and IRP2 are expressed in the kidney, with IRP1 being more distributed in the proximal tubule [45]. HIF1α is found in renal tubular cells, whereas HIF2α is restricted to renal endothelial cells and interstitial cells [47]. HIF2α promotes erythropoietin production by renal interstitial fibroblasts under hypoxic conditions, which can be inhibited by IRP1 [48]. Cultured human glomerular endothelial cells have been shown to express TfR1, FPN and DMT1 [49]. The basolateral membrane is the site of tubular iron export, and the only iron exporter that has been identified in the kidney tubules is FPN, which is present in the proximal tubules [50].

The renal reabsorption of Tf and iron plays an important role in iron regulation in the renal tubular epithelial system [11,12,51]. It has been shown that renal tubular dysfunction, as observed in patients with Fanconi syndrome, Dent disease and Lowe syndrome, results in reduced reabsorption [12,52,53]. Thus, renal tubular dysfunction can increase urinary Tf and iron excretion. In the glomeruli, transferrin-bound iron (TBI) can enter mesangial cells and endothelial cells *via* TfR1 and podocytes *via* an as yet unidentified transporter [11]. A limited iron-transferrin complex can be bound to TfR1 and megalin-cubilin complexes on the parietal membrane of proximal tubular epithelial cells by mediating the endocytosis of transferrin [54]. In addition, TfR1 and NGALR, which are correlated with apical membrane TBI uptake, are also expressed in the distal convoluted tubules and collecting ducts, respectively [55]. NTBI is currently considered as the main contributor to the pathology of iron-overload disorders [37,38]. Renal uptake of NTBI has been demonstrated in human and mouse proximal tubule cell lines and in distal tubule segments [51,56,57]. Therefore, the alteration of NTBI in kidney disease and the relationship with renal ferroptosis deserve to be noticed.

Erythropoietin (EPO) is largely produced by the kidney, and renal cell loss and inflammation-mediated inhibition of EPO synthesis in CKD lead to reduced erythropoiesis and contribute to anemia [58,59]. In glomerular disorders, glomerular leakage increases the renal filtration of plasma iron, leading to increased urinary excretion of iron and EPO, systemic iron deficiency and, ultimately, anemia [60–62]. In addition, chronic inflammation stimulates hepcidin synthesis, resulting in functional iron deficiency and anemia [63]. Therefore, the occurrence and progression of renal disease can be prevented or minimized by fully understanding the mechanisms regulating renal iron metabolism.

## 3. Inflammation and hematuria are key to iron metabolism in IgAN

**Figure 1.**
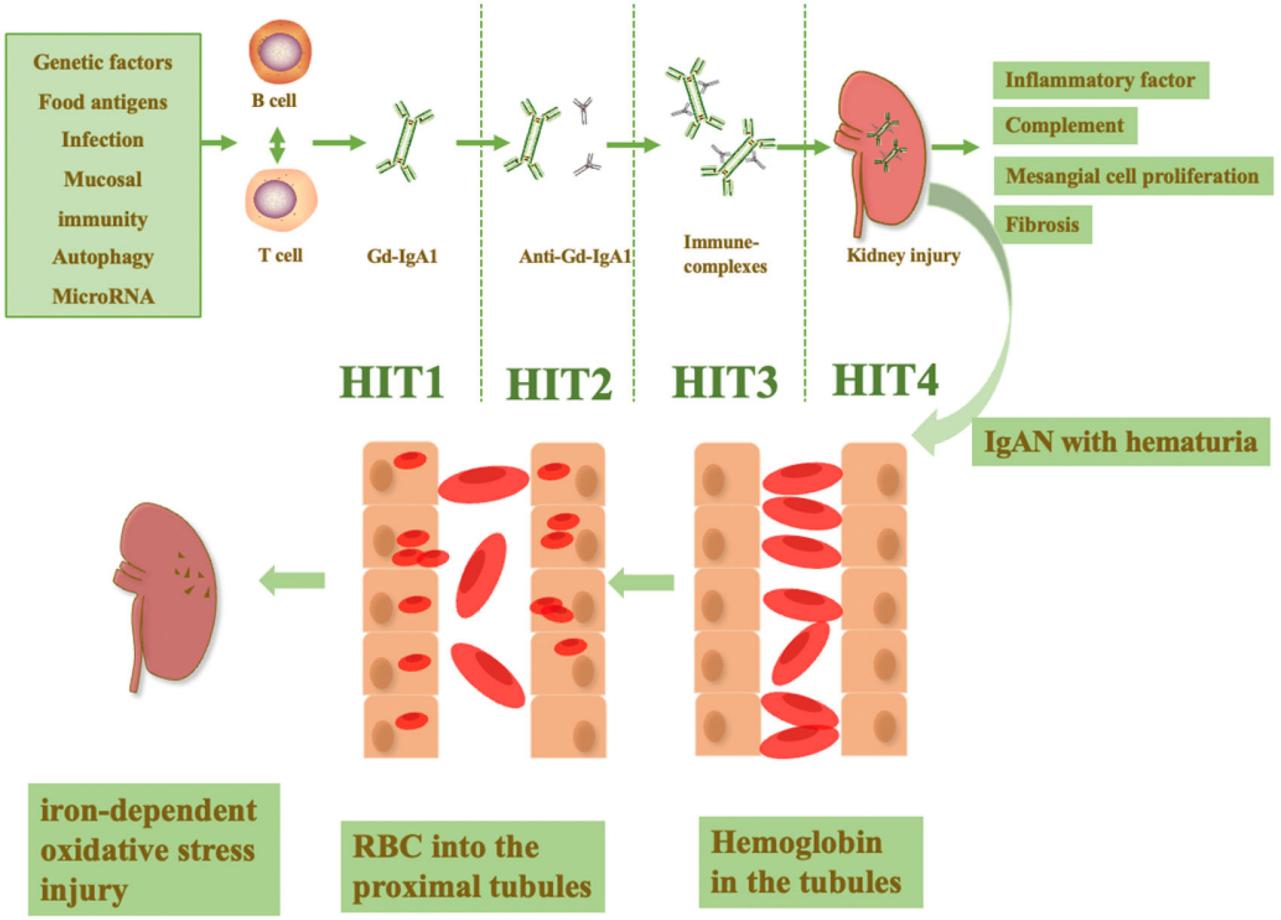
The ‘multi-hit pathogenesis’ of IgAN and hematuria in IgAN. The microbiota, genetic susceptibility, food antigens, infection, mucosal immunity, autophagy and microRNA all contribute to the pathogenesis of IgAN. The ‘multi-hit pathogenesis’ explains the formation of circulating complexes deposited in the glomerular membranes, activating mesangial cells and complement and leading to kidney injury in IgAN. In IgAN patients with hematuria, the entry of red blood cells into the proximal tubule can contribute to red blood cell uptake by the renal tubules and the accumulation of intratubular hemoglobin, leading to iron-dependent oxidative stress renal interstitial injury.

The pathogenesis of IgAN is complex and has been discovered to be associated with microbiota, genetic susceptibility, food antigens, infection, mucosal immunity, autophagy, and microRNAs [64–67]. The ‘multi-hit pathogenesis’ has been proposed to interpret the mechanism of renal damage by IgAN: the first hit is the generation of galactose-deficient IgA1 (Gd-IgA1); the second hit is the formation of IgA autoantibodies against (Gd-IgA1); the third hit autoantibodies bind to Gd-IgA1 and form a circulating immune complex that is not adequately cleared from the circulation; the fourth hit is this circulating complex depositing in the glomerular membranes, activating mesangial cells and complement and leading to kidney injury [68] (Figure 1). The ‘multi-hit pathogenesis’ is the key to the formation of inflammation in the glomerulus and cell–cell crosstalk pathways [69–72].

IgAN is mainly characterized by hematuria and proteinuria as the main clinical symptoms [73]. For a long time, we have focused more on the damage caused by proteinuria in the kidneys and neglected the damage caused by hematuria. In 1992, Nath first suggested that hematuria can lead to progressive chronic kidney disease for the following reasons: (1) glomerular disease can cause the presence of red blood cells in the urinary tract, which are phagocytosed by the renal tubules and release hemoglobin in the tubules; (2) the injection of red blood cells into the proximal tubules of rodents can contribute to the renal tubular uptake of erythrocytes, intratubular hemoglobin accumulation and tubulointerstitial disease; and (3) hemoglobin facilitates iron-dependent oxidative stress injury [26] (Figure 1). These results demonstrate impairment of IgAN by hemoglobin, which may cause renal injury mainly by promoting iron-dependent oxidative stress; thus, exploring the relationship between IgAN and iron metabolism is of more interest.

## 4. Changes in iron metabolism indicators in IgAN

Iron metabolism is integrally related to the pathogenesis, pathological diagnosis, activity assessment and prognostic evaluation of IgAN. Among these processes, TfR might be involved in the pathogenesis and progression of IgAN. To some extent, sTfR is correlated with the assessment of the activity of IgAN. sTfR and Tf may indirectly reflect the degree of IgAN pathology. Hepcidin is expressed in the kidney, has a renoprotective effect in acute kidney injury (AKI) and is associated with renal anemia but is not currently studied in IgAN. In addition, the expression of GPX4, as the core factor of ferroptosis, is reduced in IgAN, and presumably ferroptosis also occurs in IgAN.

### 4.1. TfR and IgAN

The transferrin receptor (TfR, also known as CD71) in mammals has two types, TfR1 and TfR2. TfR1 is a 97-kDa type 2 membrane protein that is expressed as a homodimer in cell membranes [74]. TfR1 mainly forms the TfR1-Tf-Fe complex to mediate the entrance of iron into cells, and its expression is regulated by the cellular iron status [75]. When iron demand increases, TfR1 is upregulated in cells that require energy or are rapidly proliferating, such as erythrocytes, osteoblasts, cancer cells and activated lymphocytes [76]. TfR2 includes two types of receptors: TfR2-α and TfR2-β[77]. TfR2-α regulates iron by enhancing hepcidin expression in hepatocytes, regulating erythropoiesis and fostering the transportation of iron to mitochondria [78]. TfR2-β modulates iron metabolism by augmenting the expression of iron transport proteins in monocytes/macrophages [78]. In 2001, Ivan C. Moura et al. first identified TfR on renal mesangial cells as the major cell surface receptor for binding to IgA1 in IgAN and found that the proliferative state of mesangial cells was associated with TfR overexpression in IgAN [79,80]. A cohort study showed that Gd-IgA1 from IgAN patients combined more efficiently with TfR than healthy controls, suggesting that the formation of immune complexes favors mesangial TfR-IgA1 interactions and can cause a 3- to 4-fold increase in TfR expression *via* positive feedback [19,20]. In 2021, Jong Hyun Jhee et al. showed that mesangial TfR is significantly associated with disease progression and may play a biologic role in IgAN [81]. Additional studies have shown that IgA1 deposition leads to TfR1 upregulation because of the direct binding of sCD89 to TfR1 on mesangial cells and that the sCD89-TfR1 interaction induces the expression of transglutaminase 2 (TGase2) on the surface of mesangial cells, which in turn upregulates TfR1 [82]. However, it was found that in addition to TfR, other antibodies such as sCD89, β-1,4-GalT1 were also expressed on the mesangial cells of IgAN which bind to IgA1, because blocking TfR does not completely eliminate mesangial cell binding to IgA [82,83]. Marijn M et al. consequently identified a new IgAN antibody, β1,4-galactosyltransferase 1 (β-1,4-GalT1), which is a receptor that binds to the Fc portion of IgA, maintaining glomerular homeostasis and interacting with IgA-TfR to share intracellular signaling pathways [83]. Moreover, Feng et al. injected ferroptosis cell membranes into mice to generate an immune response and screened 3F3-FMA antibodies from antibodies whose antigen is transferrin [84], which indicated that TfR is a receptor in ferroptosis.

In conclusion, transferrin receptors are associated with the pathogenesis of IgAN. However, whether the high expression of TfR in the glomerular mesangial region causes iron overuptake in mesangial cells and further triggers cellular injury still needs to be further investigated.

### 4.2. sTfR and IgAN

Soluble transferrin receptor (sTfR) is a fragment of TfR on the cell membrane secreted into the circulation by protease hydrolysis, which can indirectly reflect the cytosolic expression of TfR [85]. sTfR can reflect iron deficiency in anemia and inflammatory status [86]. In addition, sTfR is also a strong predictor of heart failure. A study of 287 individuals with type 2 diabetes and coronary arteriosclerotic disease, with a mean follow-up of 45 months, showed that serum ferritin and sTfR independently of other risk factors strongly predict 5-year all-cause mortality [87]. Recent studies have shown that sTfR is also linked to rheumatoid arthritis, inflammatory bowel disease, obesity and postpartum depression [88–92]. A cohort study found that the concentration of sTfR was significantly higher in the blood and urine of IgAN patients than in controls [93]. Urinary sTfR concentrations were significantly reduced when active IgAN patients were in complete remission [21]. Hence, sTfR in blood and urine can be utilized as an indicator to facilitate early diagnosis and activity assessment of IgAN patients.

### 4.3. Tf and IgAN

Transferrin (Tf) is a 76-kDa glycoprotein generated by the liver with a half-life of approximately 8 days in serum. It is the principal iron transporter protein in the body and translocates circulating iron to cells [94]. Transferrin is very similar to albumin in molecular weight but has a higher isoelectric point, and urinary transferrin consequently precedes urine albumin in glomerular disease [95,96]. In addition, urinary transferrin can predict the severity of mesangial cell proliferation and glomerulosclerosis in the early stages of potentially progressive glomerular disease [97]. Urinary Tf can be evaluated to assess cardiovascular risk [98]. A cohort study showed significant differences in urinary Tf and the urinary/plasma Tf ratio in patients with type 2 diabetes compared to healthy controls, and urinary Tf was correlated with higher carotid intima-media thickness values [98]. In a retrospective study of 514 IgAN patients, urinary Tf was positively correlated with mesangial cell hyperplasia, endothelial cell hyperplasia, tubular atrophy or interstitial fibrosis in the Oxford classification of IgAN [99]. However, these studies [97–99] were only cross-sectional, and patient follow-up information needs to be added to explore the impact of urinary Tf on the prognosis of IgAN. In conclusion, Tf is relevant to the pathological manifestations of IgAN, and whether urinary Tf can be applied to assess cardiovascular risk in chronic kidney disease needs to be further investigated.

### 4.4. Hepcidin and IgAN

**Figure 2.**
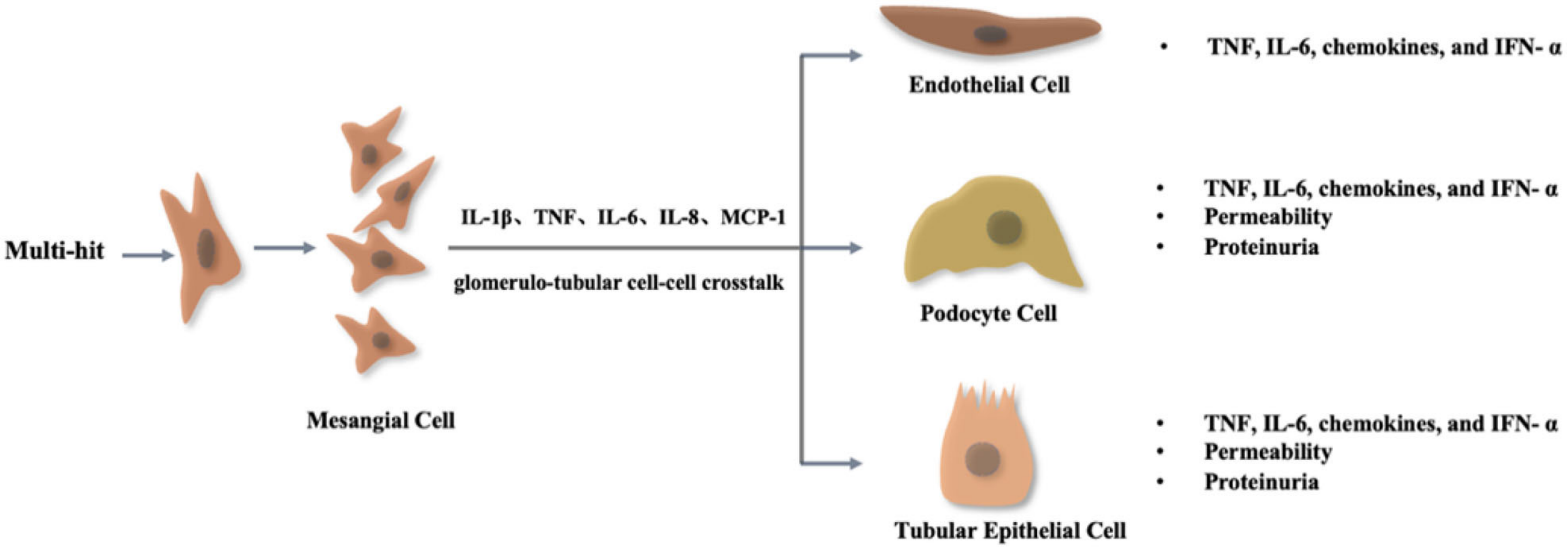
The activation of resident kidney cells contributes to chronic inflammation in IgAN. The ‘multi-hit’ hypothesis causes mesangial cells to proliferate and secrete inflammatory factors (IL-1β, TNF, IL-6, IL-8, and MCP-1). These inflammatory factors reactivate resident kidney cells by glomerulo-tubular cell–cell crosstalk pathways and produce proinflammatory chemokines responsible for perpetuating the cycle of chronic inflammation leading to kidney fibrosis.

**Figure 3.**
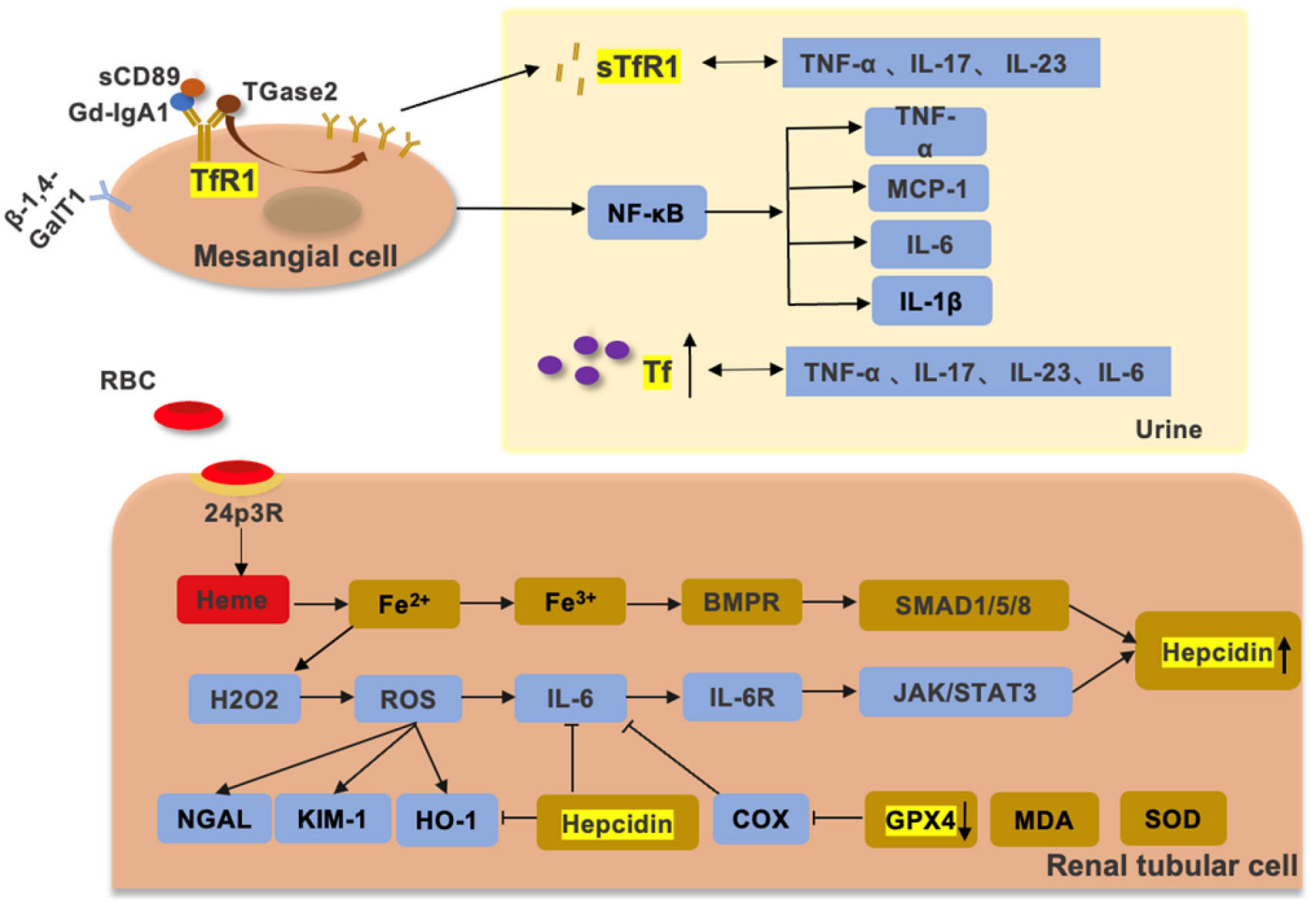
The relationship between iron metabolism, IgAN, and chronic inflammation. β-1,4-GalT, sTfR1, and CD89 are expressed by antibodies on mesangial cells in IgAN. Gd-IgA1 activates inflammation by promoting TfR1 overexpression through positive feedback to promote the secretion of IL-6, TNF-α, IL-1β and MCP-1 by the NF-κB pathway [107–109]. Overexpressed TfR1 can release sTfR1, which positively correlates with TNF-α, IL-17 and IL-23. When the glomerular filtration barrier is disrupted, Tf is increased in the urine. Tf is positively correlated with TNF-α, IL-17, IL-23, IL-6 and CRP. Ferroptosis is present in IgAN, and hemoglobin from hematuria is reabsorbed by 24p3R in renal tubular cells. Due to the tubular toxicity of hemoglobin, Fe3+ produced by the Fenton reaction promotes oxidative stress, causing increased expression of NGAL, KIM-1, HO1 and IL-6. The SMAD/BMP pathway and JAK/STAT3 pathway promote the expression of hepcidin [11,110]. GPX4 and SOD expression was decreased and MDA expression was increased in IgAN. GPX4 can suppress IL-6 by inhibiting LOX. In addition, hepcidin achieves renal protection by inhibiting IL-6 and HO-1 in hemoglobin-mediated renal tubular injury.

Hepcidin (also known as HAMP) is a 25-amino-acid peptide produced by hepatocytes that acts as a key negative regulator of small intestinal iron uptake and macrophage iron release [100,101]. Interestingly, a growing body of data suggests that hepcidin is expressed in the kidney and exerts a renoprotective effect [102,103]. Among these structures, hepcidin is preferentially expressed in the distal renal tubules, mainly in the thick segment of the ascending branches of the renal medulla but not in the proximal tubules [102]. The kidney was shown to defend against iron-mediated renal injury by reabsorbing hepcidin in the proximal tubule and synthesizing it in the distal tubule [104]. Mohammad et al. found that the hepcidin/FPN axis exerts is of crucial value in regulating renal and systemic iron homeostasis under iron overload in mouse kidneys [104]. In addition, hepcidin is closely related to renal anemia and shows a renoprotective effect in AKI [103,105,106]. Van Swelm et al. found that hepcidin reduces renal tubular damage from hemoglobin in mice with AKI [103,105] (Figure 2 and 3).

Persistent microscopic hematuria was shown to be a risk factor for IgAN progression, and in a study of up to 1 million Israeli middle-aged adults with primary glomerular disease, patients with primary glomerular disease exhibiting persistent microscopic hematuria had a markedly increased risk of ESRD compared to controls [111]. In a retrospective study of 1333 cases of IgAN in China, hematuria was found to still be an independent risk factor for renal composite endpoint events, and patients with hematuria remission occurring within 6 months of diagnosis had significantly lower renal composite endpoint events [111]. In a cohort study of 112 patients with IgAN, hematuria remission was identified as having a statistically significant favorable impact on IgAN outcomes, with a significantly higher proportion of patients with persistent hematuria reaching the renal composite endpoint event than patients with mild or negative hematuria [112]. Forty-six percent of patients with negative hematuria had a change in the rate of decline of renal function from −6.45 ± 14.66 to −0.18 ± 2.56 mL/min/1.73 m2/year [112].

In short, hepcidin in the kidney has a beneficial effect on hemoglobin-induced tubular injury, and hemoglobin in IgAN causes tubular injury by promoting iron-dependent oxidative stress. However, hepcidin has not been studied in IgAN at this time. Therefore, exploring the expression of hepcidin in IgAN patients and verifying the protective role of hepcidin in IgAN probably provides a basis for the future treatment of IgAN hematuria (Table 1).

**Table 1.** The relationship between iron metabolism and IgAN.

Iron index		Expression	Induction	Function	Association with IgAN
TfR	TfR1	Rapidly proliferating cells: erythrocytes, hepatocytes, osteoblasts, cancer cells and activated lymphocytes [113]	Cellular iron levels [114]; Up-regulated upon mitogenic stimulation [115]	Cellular uptake of iron by TfR1-Tf-Fe [75]; Endosomal acidification leads to iron release [116]; As a lipid sensor regulate mitochondrial fusion [117]; Microbial infection [118]; Ferroptosis marker [84]	As the major cell surface receptor for binding to IgA1 in IgAN [20,79]; Associated with the progression of IgAN [81]
	TfR2	Hepatocytes and erythroid cells [119]	Cellular iron levels [120]	Mediates cellular uptake of transferrin-bound iron in a non-iron dependent manner [120]; May be involved in iron metabolism, hepatocyte function and erythrocyte differentiation [119];	No research
sTfR		A fragment of TfR on the cell membrane [85]	Iron deficiency [86]	Reflect iron deficiency and inflammatory status [86]	A potential biomarker of IgAN [21]; Inhibited IgA1 binding (>50%) [19]; A marker of erythropoiesis in dialysis patients [121]
Tf		liver [94]	Iron deficiency [122]	Translocate circulating iron to cells [94]	Associated with Oxford classification of IgAN; Predicts ESRD in T_2_DM patients [123]
Hepcidin		Liver, heart, brain, lung, tonsils, salivary gland, trachea, prostate gland, adrenal gland, thyroid gland and kidney [104,124]	Erythropoiesis, anemia, and iron overload [100]	Main circulating regulator of iron [125,126]; Antimicrobial activity [127]	Regulated intrarenal iron handling at the distal nephron [128]; Defends against iron-mediated renal injury in AKI [104]; Renal anemia [129,130]
GPX4		Testis and platelets [131]; Cells undergoing lipid hydroperoxide toxicity: cancer [132], kidney [133], neuronal [134], gut [135] and liver [136]	Lipid oxidation [137]	Antioxidant peroxidase [132]; Against ferroptosis [131]	Related to the treatment of AKI [133,138], ADPKD [139] and DKD [140].
HO-1		Macrophages in the spleen and liver [33]; Most cells in response to pro-oxidants [141]	Iron-recycling; Upregulated by pro-oxidants [142]	Decomposes heme to provide iron [33]; Antioxidants [141]	A risk factor of IgAN [143]

T_2_DM: type 2 diabetes mellitus; AKI: acute kidney injury; ADPKD: autosomal dominant polycystic kidney disease; DKD: diabetic kidney disease.

### 4.5. Ferroptosis and IgAN

Ferroptosis is an iron-dependent, novel form of programmed cell death that is distinct from apoptosis, cell necrosis and cell autophagy [144]. Ferroptosis is caused by unrestricted lipid peroxidation, eventually contributing to irreversible plasma membrane damage [145]. Ferroptosis includes three common metabolic pathways: iron ion metabolism, lipid peroxidation reactions and glutathione metabolism. When ferroptosis occurs, a large amount of free Fe^2+^ accumulates in the cell, which is highly oxidizing and easily reacts with H2O2 to produce hydroxyl radicals that can cause oxidative damage to DNA, proteins and membrane lipids, which promotes the occurrence of lipid peroxidation, damages the cell membrane and leads to cell death [146]. In ferroptosis, lipid peroxidation damage leads to the oxidative degradation of two important biofilm components, polyunsaturated fatty acids (PUFAs) and phosphatidylethanolamine (PE). The disruption of PUFA and PE structures in biofilms can result in cell rupture death and interference with cellular functions, such as oxidative phosphorylation, mitochondrial production and autophagy [147,148]. Currently, studies have confirmed that GPX4 is a central regulator of ferroptosis, and the depletion of glutathione (GSH) leads to GPX4 inactivation, which increases intracellular lipid peroxidation and ferroptosis [149].

The three metabolic pathways of ferroptosis can also occur in IgAN. In iron ion metabolism, the main manifestation is the abnormal deposition of iron ions in renal tissues. A retrospective study showed that in renal tissues, the amount of iron deposition directly correlated with the incidence of mean arterial pressure, serum creatinine level, urinary protein excretion and hematuria in patients with kidney disease [150]. A case report described an IgAN patient with acute kidney injury and massive hematuria, whose Prussian blue iron staining of renal biopsy tissue suggested acute tubular necrosis caused by massive deposits of iron-containing heme in the renal tubules; the authors speculated that iron-containing heme deposits may be a valid indicator of the pathophysiology of AKI associated with sarcoid hematuria [151]. Superoxide dismutase (SOD) and malondialdehyde (MDA) are involved in the lipid peroxidation process, where SOD and VitE attenuate the lipid peroxidation metabolic process and MDA is a toxic substance produced by the lipid peroxidation metabolic process [152]. In a cohort study, the activity of SOD and VitE in the serum was significantly lower and the level of MDA was significantly higher of IgAN patients than in healthy controls [153]. In patients with IgAN, MDA levels were significantly higher in the moderate pathology group than in the mild pathology group, and SOD activity was lower in the moderate pathology group than in the mild pathology group [153]. Compared to controls, the expression of GPX4 was significantly lower in the renal tissue of patients with IgAN [154]. Recent studies have shown that TfR is a specific receptor in ferroptosis [84], but the relationship between TfR and ferroptosis in IgAN still needs to be studied.

Based on the three metabolic pathways mentioned above, ferroptosis can be hypothesized to play an important role in the pathogenesis of IgAN and disease progression. Many drugs have been found to have a therapeutic effect in the clinic to reduce ferroptosis, including licorice, quercetin, apigenin and vitamin K [155–158]. In short, mitigating ferroptosis may have new value in the treatment of IgAN.

### 4.6. HO-1 and IgAN

Two functional isoforms of heme oxygenase exist in mammalian cells: HO-1 and HO-2. HO-2 is expressed in the brain, testis, cardiovascular and liver and can balance iron and redox metabolism as well as cellular messaging [159–161]. By contrast, HO-1 is a stress-inducible isozyme [162]. Under homeostatic conditions, HO-1 is constitutively expressed in iron-recycling macrophages in the spleen and liver and certain tolerogenic immune cells [163,164]. However, HO-1 is highly upregulated by most cells in response to free heme and many other pro-oxidants to provide protection against oxidative damage [141,142,165]. Micro and/or macroscopic hematuria is a typical symptom of IgAN that suggests potential induction of HO-1 in the glomeruli [166]. The HO-1 gene promoter length polymorphism was an important risk factor for mortality in IgAN [143].

## 5. The interconnection of iron metabolism, chronic inflammation and IgAN

Iron metabolism is connected with the occurrence of chronic inflammation in IgAN, and both promote each other. TfR enhances the proliferation of mesangial cells and inflammatory factor production in IgAN. Tf correlates with human immunity. When Tf decreases, bacterial, chlamydial, viral and other pathogenic microorganism infections can be increased. Tf, sTfR and inflammatory factors are positively correlated in IgAN. IL-6 is increased in IgAN tissue, blood, and urine, and IL-6 promotes hepcidin expression through the activation of the JAK/STAT signaling pathway. In addition, the reduction in GPX4 expression in IgAN may facilitate ferroptosis and interfere with inflammatory homeostasis.

### 5.1. Chronic inflammation is the essence of IgAN

Acute inflammation is marked by infiltrating leukocytes, but chronic inflammation is a prolonged, dysregulated and maladaptive response that involves active inflammation, tissue destruction and attempts at tissue repair [167,168]. In CKD, chronic inflammation is characterized by the recruitment of leukocytes and activation of resident kidney cells, including mesangial cells, endothelial cells, tubular epithelial cells, and podocytes, which exhibit a proinflammatory phenotype that eventually leads to kidney fibrosis and loss of kidney function [169].

In IgAN, the ''multi-hit’' hypothesis elaborates on the deposition of circulating immune complexes on mesangial cells that cause the secretion of extracellular matrix and release of proinflammatory and profibrotic cytokines [170–173], which leads to the stimulation of mesangial cell proliferation and recruitment of inflammatory cells into the glomerulus [174]. Inflammatory mediators also modify gene expression in podocytes, tubular epithelial cells, and endothelial cells, resulting in podocyte, tubulointerstitial and endothelial damage by glomerulo-tubular cell–cell crosstalk pathways in IgAN [170,175–178]. The above statements illustrate that IgAN is a typical chronic inflammatory disease.

### 5.2. TfR, chronic inflammation and IgAN

The overexpression of TfR on IgAN mesangial cells may promote the proliferation and inflammation of mesangial cells *via* positive feedback. Indirect immunofluorescence confirmed that TfR on IgAN mesangial cells preferentially binds to the poly IgA1 complex (pIgA1). The combination of pIgA1 and TfR enhanced the proliferation of mesangial cells and induced IL-6 and TGF production [19,20,179]. Additionally, a cohort study of 288 patients with IgAN found that TfR mRNA expression levels were higher in nonprogressive IgAN patients than in controls, and these differences were more pronounced in the progressive group. Furthermore, siRNA silencing of TfR significantly reduced IL-6, TNF-α, and MCP-1 expression [81] (Figure 3). Thus, TfR is closely correlated with mesangial cell activation and chronic inflammation in IgAN.

### 5.3. sTfR, chronic inflammation and IgAN

sTfR is mainly positively associated with the severity of iron deficiency. Under conditions of iron deficiency, a compensatory increment of TfR on the cell surface increases the cellular uptake of iron on the one hand and sTfR production on the other [180]. A prospective study showed that the levels of serum TNF-α, IL-17, and IL-23 in hemodialysis patients were positively correlated with Tf and sTfR [181] (Figure 3). This finding suggests a close relationship between abnormal iron metabolism and the microinflammatory response during hemodialysis, in which disturbances in the inflammatory response can affect iron metabolic processes and cause iron deficiency to varying degrees [181]. The current study confirms that sTfR levels in IgAN serum are increased and can be utilized for the assessment the activity of IgAN [21], but whether sTfR is involved in the inflammatory status of IgAN patients *in vivo* still needs to be further investigated.

### 5.4. Tf, chronic inflammation and IgAN

Tf is decreased when liver dysfunction and inflammation appear [78]. Almost all microbial pathogens need iron to successfully infect their mammalian hosts. Tf can provide a defense against systemic infections by inhibiting the binding of iron to potential pathogens, including coagulase-positive Staphylococcus aureus, Staphylococcus epidermidis, Pseudomonas aeruginosa, Bacillus anthracis, Chlamydia, SARS-CoV2 and other pathogenic microorganisms [182–184]. A retrospective study of 117 patients with primary glomerulonephritis showed a strong relationship between urinary Tf concentration and tubulointerstitial damage, where higher urinary Tf concentrations were found to be associated with more severe tubulointerstitial damage [185].

Studies on Tf and inflammatory factors in IgAN are currently lacking. Moreover, urinary Tf excretion is increased in patients with IgAN, and whether this increase further affects iron homeostasis in the circulatory system needs to be further investigated. In addition, Tf has anti-infective and immune defense effects, and whether IgAN patients are more susceptible to infection when they have less Tf in the circulation should be of interest.

### 5.5. Hepcidin, chronic inflammation and IgAN

Hepcidin is modulated by iron availability, erythropoiesis and inflammatory status [100]. Hepcidin expression increases under iron overload and inflammation and reduces under iron deficiency and hypoxia [186]. However, many of the current studies on hepcidin and inflammation are in the hepatocytes or AKI [187–189]. In IgAN, immune damage in the mesangial region promotes IL-6 secretion [171,190], and the tubular damage caused by hematuria also leads to an increase in IL-6 [26,191]. Thus, we speculate that IL-6 may promote hepcidin production by activating the JAK/STAT signaling pathway in IgAN (Figure 3).

### 5.6. Ferroptosis, chronic inflammation and IgAN

The relationship between ferroptosis and inflammation is complementary and mutually reinforcing. When ferroptosis occurs, a large amount of free Fe^2+^ accumulates in the cell, which is highly oxidizing and easily reacts with H2O2 to produce hydroxyl radicals (i.e., elevation of reactive oxygen species [ROS])[146]. Persistent low-grade inflammation and ROS are partners in crime, and the two are positive feedback loops in which one amplifies the other, ultimately leading to the progression of kidney disease [174]. Lipoxygenase (LOX) and cyclooxygenase (COX) are important factors in the metabolic process of inflammation, and GPX4 can suppress inflammation by inhibiting LOX and COX [192] (Figure 3). Therefore, the significantly reduced GPX4 expression in the renal tissues of IgAN patients may be relevant to the chronic inflammatory state of IgAN patients. Ferroptosis inhibitors have been shown to be effective in alleviating the inflammatory response, but the value of ferroptosis inhibitors in IgAN needs to be further explored.

### 5.7. HO-1, chronic inflammation and IgAN

HO-1 is upregulated during inflammation to protect against the potentially harmful effects of reactive oxygen species and pro-inflammatory cytokines [193]. Interestingly, HO-1 is also an important immune regulator in the macrophage population, associated with promoting anti-inflammatory M2 macrophage polarization and limiting the pro-inflammatory activity of M1 macrophages [194–196]. The HO-1 system allows macrophages to protect tissues from oxidative damage, a role that is associated with an important beneficial role in atherosclerosis, ischemic injury and kidney disease [197–199]. Although HO-1 is broadly anti-inflammatory in nature, it is not always beneficial and is associated with deleterious outcomes in diseases such as cancer, obesity, and chronic infections [200–202]. Thus, induction or inhibition of HO-1 in macrophages may have therapeutic effects, depending on the disease context. HO-1 expression was increased in IgAN because of its anti-inflammatory effects [143,166] and therefore may have a renoprotective role in IgAN. However, the specific application value of HO-1 in IgAN still needs to be further explored.

## 6. Therapeutic value of iron metabolism indicators in IgAN

The management of IgAN is focused on nonimmunosuppressive strategies. This strategy encompasses rigorous blood pressure control, optimal inhibition of the RAS, and lifestyle modification, including weight reduction, exercise, smoking cessation, and dietary sodium restriction [203].

TfR1 is required to regulate iron metabolism in high-metabolism cells in the human body, such as erythrocytes, hepatocytes, and lymphocytes [113], targeting TfR1 is highly likely to affect cellular iron metabolism. sTfR is the shedding product of TfR [85], and sTfR can essentially reflect the treatment effect of IgAN to some extent. Tf is associated with early kidney injury [96] and may reflect kidney recovery after treatment. Hepcidin has a central role in iron regulation, modulating the hepcidin-ferroportin axis by agonists/antagonists [100]. Hepcidin agonists may be able to prevent and treat iron overload. In mouse models, mini-hepcidin has been shown to control iron overload [204]. Hepcidin antagonists can inhibit hepcidin activity to form the basis of treatment of anemia characterized by iron restriction, such as anemia of inflammation and cancer [205]. Therefore, we speculate that the appropriate dose of hepcidin agonist that can be used in renal disease when excessive hematuria causes iron pigmentation still needs some exploration. When renal disease is complicated by anemia, the use of hepcidin antagonists is reasonable, but attention needs to be paid to the detection of iron concentrations to prevent iron overload. Iron storage, oxidative damage, and inflammation increase with kidney aging, iron dysregulation may be a causal factor in the renal dysfunction that accompanies aging [11,206]. Iron chelation improves renal function in animal models of renal lesions, as well as in humans with renal dysfunction [207,208]. Desferrioxamine (DFO) is an iron chelator approved for the treatment of iron overload in humans [209–211].

## 7. Conclusion

In summary, iron metabolism-related indicators are related to the pathogenesis, pathological diagnosis, activity and prognostic assessment of IgAN. However, there are still some iron metabolism pathways that have not been elucidated for different kidney cells. IgAN can cause disorders of iron metabolism, and disorders of iron metabolism can aggravate IgAN progression. Iron metabolism is closely related to chronic inflammation in IgAN. The hepcidin-ferroportin axis modulated by agonists/antagonists represents an attractive future target for novel preventive or therapeutic strategies for disorders of iron metabolism in IgAN.
